# Pirfenidone modulates macrophage polarization and ameliorates radiation‐induced lung fibrosis by inhibiting the TGF‐β1/Smad3 pathway

**DOI:** 10.1111/jcmm.16821

**Published:** 2021-07-29

**Authors:** Hangjie Ying, Min Fang, Qing Qing Hang, Yamei Chen, Xu Qian, Ming Chen

**Affiliations:** ^1^ Institute of Basic Medicine and Cancer (IBMC) The Cancer Hospital of the University of Chinese Academy of Sciences (Zhejiang Cancer Hospital Chinese Academy of Sciences Hangzhou China; ^2^ Zhejiang Key Laboratory of Radiation Oncology Zhejiang Cancer Hospital Hangzhou China; ^3^ The Department of Thoracic Radiotherapy Zhejiang Cancer Hospital Hangzhou China; ^4^ The Second Clinical Medical College of Zhejiang Chinese Medical University Hangzhou China; ^5^ The Department of Clinical Laboratory Zhejiang Cancer Hospital Hangzhou China

**Keywords:** ionizing radiation, macrophages, pirfenidone, radiation‐induced lung fibrosis, transforming growth factor‐β1

## Abstract

Radiation‐induced lung injury (RILI) mainly contributes to the complications of thoracic radiotherapy. RILI can be divided into radiation pneumonia (RP) and radiation‐induced lung fibrosis (RILF). Once RILF occurs, patients will eventually develop irreversible respiratory failure; thus, a new treatment strategy to prevent RILI is urgently needed. This study explored the therapeutic effect of pirfenidone (PFD), a Food and Drug Administration (FDA)‐approved drug for (IPF) treatment, and its mechanism in the treatment of RILF. In vivo, C57BL/6 mice received a 50 Gy dose of X‐ray radiation to the whole thorax with or without the administration of PFD. Collagen deposition and fibrosis in the lung were reversed by PFD treatment, which was associated with reduced M2 macrophage infiltration and inhibition of the transforming growth factor‐β1 (TGF‐β1)/Drosophila mothers against the decapentaplegic 3 (Smad3) signalling pathway. Moreover, PFD treatment decreased the radiation‐induced expression of TGF‐β1 and phosphorylation of Smad3 in alveolar epithelial cells (AECs) and vascular endothelial cells (VECs). Furthermore, IL‐4–induced M2 macrophage polarization and IL‐13–induced M2 macrophage polarization were suppressed by PFD treatment in vitro, resulting in reductions in the release of arginase‐1 (ARG‐1), chitinase 3‐like 3 (YM‐1) and TGF‐β1. Notably, the PFD‐induced inhibitory effects on M2 macrophage polarization were associated with downregulation of nuclear factor kappa‐B (NF‐κB) p50 activity. Additionally, PFD could significantly inhibit ionizing radiation‐induced chemokine secretion in MLE‐12 cells and consequently impair the migration of RAW264.7 cells. PFD could also eliminate TGF‐β1 from M2 macrophages by attenuating the activation of TGF‐β1/Smad3. In conclusion, PFD is a potential therapeutic agent to ameliorate fibrosis in RILF by reducing M2 macrophage infiltration and inhibiting the activation of TGF‐β1/Smad3.

## 1 INTRODUCTION

Radiotherapy is a standardized treatment for thoracic tumours such as lung cancer,^1^ oesophageal cancer,^2^ malignant lymphoma^3^ and breast cancer.^4^ Clinical data show that the incidence of radiation‐induced lung injury (RILI) is 5%–25% after radiation therapy in patients with lung cancer, followed by those with mediastinal lymphoma (5%–10%) and breast cancer (1%–5%).^5^ There are two clinical manifestations of RILI: radiation pulmonary (RP) and radiation‐induced lung fibrosis (RILF). To date, corticosteroids are mainly used to control RP in the clinic, while RILF leads to progressive alveolar structural disorders and irreversible pulmonary fibrous tissue remodelling, which eventually causes respiratory failure. There is currently no effective drug available to reverse RILF.^6^

Macrophages are highly malleable, and their functional phenotypes depend on different microenvironments. After exposure to Toll‐like receptor (TLR) ligands, macrophages experience a phenotype known as classically activated macrophages or M1 macrophages, which can produce high levels of pro‐inflammatory cytokines, such as tumour necrosis factor‐α (TNF‐α), interleukin‐6 (IL‐6) and inducible nitric oxide synthase (iNOS). In contrast, alternately activated macrophages or M2 macrophages are produced in response to cytokines such as IL‐4 and IL‐13 and highly express M2 macrophage‐associated inflammatory factors, such as chitinase 3‐like 3 (YM‐1) and arginase‐1 (ARG‐1).^7^ M1 macrophages have been shown to prevent the development of pulmonary fibrosis, and M2 macrophages are the most prominent type of macrophage in pulmonary fibrosis.^8^ Therefore, the balanced transition from M1 macrophages that promote inflammation to M2 macrophages that promote fibrosis and wound healing is one of the important reasons for the development of pulmonary fibrosis after radiation.

Pirfenidone (PFD) is a multipotent pyridone analog that was discovered by the MARNAC Company in 1974. The antifibrotic effect of PFD was first found in a bleomycin‐induced idiopathic pulmonary fibrosis (IPF) animal model in 1995.^9^ Later, studies have shown that PFD could inhibit fibrosis by downregulating the expression of fibrogenic growth factors, inhibiting the production and release of inflammatory cytokines and reducing the occurrence of lipid peroxidation and oxidative stress injury.^10^, ^11^, ^12^, ^13^, ^14^ PFD has a significant inhibitory effect on pulmonary fibrosis and fibrosis in other organs and is a broad‐spectrum antifibrotic drug. A number of phase III clinical studies have shown that PFD can significantly delay the decline in forced vital capacity (FVC) in patients with IPF and significantly reduce the mortality of IPF.^15^, ^16^ Based on these data, PFD became the first drug approved by the FDA to treat IPF in 2014. The pathological and physiological process of RILF is similar to that of IPF and involves the early inflammatory reaction, lung parenchymal injury, alveolar repair and interstitial fibrosis. A previous study reported that PFD could downregulate the expression of transforming growth factor‐β1 (TGF‐β1) in lung tissue, leading to the inhibition of RILF progression, but the deeper mechanism has not been elucidated.^17^

In the present study, we investigated the protective effects of PFD on RILF in vivo and in vitro. We demonstrated that PFD could reduce the polarization of M2 macrophages and inhibit the activation of TGF‐β1/Smad3 signalling of alveolar epithelial cells (AECs) and vascular endothelial cells (VECs) by ionizing radiation. PFD was also involved in the regulation of AECs and macrophages. Thus, PFD has therapeutic potential for patients with RILF.

## 2 MATERIALS AND METHODS

### 2.1 Animals and reagents

10‐ to 12‐week‐old inbred C57BL/6 female mice (body weight 18–20 g) were provided by Shanghai Xipuer Bikai Experimental Animal Co., Ltd. (production licence: SCXK 2013–0016) and housed in the SPF animal breeding room of the Experimental Animal Center of Zhejiang Chinese Medical University. The mice were housed six per cage under standard conditions and had free access to food and water. The mice were transferred in and out of the SPF animal room using dedicated sealed transfer boxes with air filtration.

### 2.2 Irradiation and PFD treatment

40 female C57BL/6 mice were randomized into four groups: the negative control (NC, normal saline) group, radiation alone (RT) group, PFD alone (PFD group) and radiation plus PFD (RT + PFD) group. For radiation exposure, the mice were anaesthetized using sodium pentobarbital (40 mg/kg, intraperitoneally) and received a single 50 Gy dose of whole‐lung X‐ray delivered by a small animal radiation research platform (4 Gy/min; SSD = 333 mm; XStrahl). Pirfenidone was obtained from Beijing Kangdini Pharmaceutical Co., Ltd. and dissolved in 0.5% carboxymethyl cellulose solution (CMC, vehicle) at a concentration of 20 mg/ml, which was given orally by gavage at a dose of 300 mg/kg/day every day based on published data.^18^, ^19^

### 2.3 Histology

All mice were fixed on the operating table and euthanized by femoral artery exsanguination at day 150 under sodium pentobarbital anaesthesia (40 mg/kg, intraperitoneally), and all efforts were made to minimize animal suffering. The right lung tissues were stored at −80°C for qRT‐PCR and Western blot analysis, and the left lung tissues were fixed in 4% paraformaldehyde, dehydrated and embedded in paraffin. Then, the lung tissues were sectioned into 4‐μm slices and stained with HE. Masson's trichrome was used to evaluate fibrosis based on 10 fields of view in each section. Five section was examined per lung, and five fields were randomly selected for each section. The severity of fibrosis in each field of the lung was assessed as the mean severity score using a semiquantitative grading system described by Ashcroft et al.^20^

After deparaffinization in xylene, hydration with graded alcohol and antigen retrieval, the tissue sections were placed in 3% hydrogen peroxide (H_2_O_2_) for 10 min at room temperature to inactivate endogenous peroxidases. The slides were washed three times in PBS, blocked with 2% bovine serum albumin (BSA, cat. no. B2064, Sigma) for 30 min at room temperature and incubated with primary antibodies against collagen I (1:200, cat. no. ab34710, Abcam), collagen IV (1:200, cat. no. ab6586, Abcam), CD79b (1:200, cat. no. 134147, Abcam), CD68(1:200, cat. no. 31630, Abcam) and CD3 (1:200, cat. no. Ab16669, Abcam) at 4°C overnight. The slides were then washed with PBS and incubated with HRP‐conjugated secondary antibodies (1:200, Beyotime) for 60 min at 37°C. The slides were then washed in PBS three times, followed by detection with Dako REALTM EnVisionTM (DAB, cat. no. PW017, Sangon Biotech) and counterstaining with haematoxylin.

For immunohistochemical (IHC) scoring, positive reactions were defined as those showing brown signals. One section was examined per lung, and five fields were randomly selected and observed under a light microscope. The intensity was scored as follows: 0: negative; 1: weak; 2: moderate; and 3: strong. The frequency of positive cells was defined as follows: 0: less than 5%; 1: 5%–25%; 2: 26%–50%; 3: 51%–75%; and 4: greater than 75%. The IHC total score was calculated as the product of the intensity and frequency scores (0–12) and was performed by trained scorers blind. After whole section examination, the IHC score was calculated as the mean total IHC score of all fields.

### 2.4 Immunofluorescence staining of lung tissue

Immunofluorescence (IF) analysis was performed on 4‐μm‐thick lung sections that had been dewaxed with xylene and hydrated using sequential ethanol (100%, 95%, 85% and 75%) and distilled water. Antigen retrieval was performed by heating the sections in 0.1% sodium citrate buffer (pH 6.0). Then, the specimens were washed with PBS and blocked with 10% FBS to eliminate nonspecific fluorescence. Immunofluorescence staining was performed using antibodies against CD68 (1:200, cat. no. 31630, Abcam) and CD163 (1:200, cat. no. 182422, Abcam), and the cell preparations were incubated with DyLight 488/647‐labelled secondary antibodies (1:200, cat. no. 150077/ab150115, Abcam).

### 2.5 Quantitative reverse transcription‐polymerase chain reaction

Total RNA was extracted with RNAiso Plus reagent (cat. no. 108–95–2, Takara). qRT‐PCR was performed using SYBR^®^ Premix Ex Taq II (cat. no. RR820A, Takara), after reverse transcribing 1 μg RNA with PrimeScript™ RT Master Mix (cat. no. RR036A‐2, Takara). qRT‐PCR analysis of the resulting cDNA was performed in triplicate with gene‐specific primers on a 7500 Fast Real‐time PCR system (Applied Biosystems, Thermo Fisher Scientific). The qRT‐PCR was carried out with the following conditions: denaturation at 94°C for 2 min, followed by 40 cycles of 94°C for 15 s and 62°C for 40 s. Gene expression levels were normalized to β‐actin by the 2^−ΔΔCt^ method and were determined relative to control samples. The primers for qRT‐PCR are listed in Table 1.

**TABLE 1:** Primer sequences used to quantitate gene expression

Gene	Sequence (5′ to 3′)
TNF‐α	Forward: AAGGCCGGGGTGTCCTGGAG
	Reverse: AGGCCAGGTGGGGACAGCTC
iNOS	Forward: CCCTTCCGAAGTTTCTGGCAGCAGCG
	Reverse: GGCTGTCAGAGCCTCGTGGCTTTGG
Arg−1	Forward: TCATGGAAGTGAACCCAACTCTTG
	Reverse: TCAGTCCCTGGCTTATGGTTACC
YM−1	Forward: GGATGGCTACACTGGAGAAA
	Reverse: AGAAGGGTCACTCAGGATAA
CCL2	Forward: TTAAAAACCTGGATCGGAACCAA
	Reverse: GCATTAGCTTCAGATTTACGGGT
CXCL10	Forward: AAGTGCTGCCGTCATTTTCT
	Reverse: TTCATCGTGGCAATGATCTC
CXCL16	Forward: CCTTGTCTCTTGCGTTCTTCC
	Reverse: TCCAAAGTACCCTGCGGTATC
CXCL5	Forward: TGCGTTGTGTTTGCTTAACCG
	Reverse: CTTCCACCGTAGGGCACTG
CXCL1	Forward: CTGGGATTCACCTCAAGAACATC
	Reverse: CAGGGTCAAGGCAAGCCTC
GM‐CSF	Forward: GGCCTTGGAAGCATGTAGAGG
	Reverse: GGAGAACTCGTTAGAGACGACTT
β‐Actin	Forward: GCATTGCTGACAGGATGCAG
	Reverse: CCTGCTTGCTGATCCACATC

### 2.6 Western blotting

Protein samples (40 μg) were mixed with SDS‐PAGE loading buffer and boiled at 100°C for 5 min. After electrophoresis, the proteins were transferred to PVDF membranes (cat. no. IPVH00010, Millipore Biotechnology). The membranes were blocked with 5% BSA for 60 min at room temperature. The membranes were incubated with primary antibodies against ARG‐1 (1:1000, cat. no. ab91279, Abcam), YM‐1 (1:1000, cat. no. ab192029, Abcam), TGF‐β1 (1:1000, cat. no. ab92486, Abcam), p‐Smad3 (1:1000, cat. no. ab52903, Abcam), Smad3 (1:1000, cat. no. ab40854, Abcam), NF‐κB p50 (1:1000, cat. no. Sc‐8414) and β‐actin (1:5000, cat. no. A2228, Sigma) in 5% BSA overnight at 4°C. Afterwards, membranes were washed three times with TBS containing 0.5% Tween 20 (TBST) for 10 min each time at room temperature and then incubated with secondary antibodies (1:5000, cat. no. ab150077, Abcam or 1:5000, cat. no. ab150113, Abcam) for 60 min at room temperature. After three additional washes with TBST, immunoreactive bands were visualized using enhanced chemiluminescent reagent (ECL, cat. no. WBKLS0500, Millipore Biotechnology) and quantified by ImageJ V1.8.0 (National Institutes of Health) analysis software.

### 2.7 Cell culture

Bone marrow cells were obtained by flushing femurs from 6‐ to 8‐week‐old mice and differentiating the cells (7 days) in Dulbecco's modified Eagle's medium (DMEM, HyClone) supplemented with 30 ng/ml macrophage colony‐stimulating factor (M‐CSF, PeproTech), 10% foetal bovine serum (FBS, Gibco) and antibiotics (Wonder Biotech).

The mouse macrophage line (RAW264.7 cells) was purchased from the Cell Bank of the Chinese Academy of Sciences. RAW264.7 cells were maintained in DMEM supplemented with 10% FBS and antibiotics. Cell cultures were maintained at 37°C in a humidified atmosphere containing 5% CO_2_. The medium was changed every 3 days until the culture reached 90% confluence. For experiments, cells were suspended in culture medium at a density of 1 × 10^6^ cells/ml. Cells at passages 3–5 were used for all experiments.

MLE‐12 cells and PMVECs were obtained from the Cell Bank of the Chinese Academy of Sciences. MLE‐12 cells and PMVECs were maintained in DMEM supplemented with 10% FBS and antibiotics. Cell cultures were maintained at 37°C in a humidified atmosphere containing 5% CO_2_. The medium was changed every 3 days until the culture had reached 90% confluence. For experiments, cells were suspended in culture medium at a density of 1 × 10^6^ cells/ml. Cells at passages 3–5 were used for all experiments.

### 2.8 Macrophage polarization and PFD treatment

Pirfenidone was dissolved in dimethyl sulfoxide (DMSO, Sigma) and used at final concentrations of 1, 10, 100 or 1000 µg/ml (The DMSO was used in the 0 PFD as control.).

RAW264.7 cells and bone marrow‐derived macrophages (BMDMs) were cultured in DMEM supplemented with PFD for 24 h before treatment with chemokines to promote macrophage polarization. After 24 h of PFD incubation, the cells were stimulated with 10 ng/ml IL‐4 (PeproTech) and 10 ng/ml IL‐13 (PeproTech) to promote M2 polarization. After an additional 24 h, cell supernatants were collected for ELISA analysis, and the cells were washed twice with PBS and harvested in RIPA or RNAiso Plus reagent for subsequent analysis.

### 2.9 MLE‐12 and PMVECs and PFD treatment

MLE‐12 cells and PMVECs were cultured in DMEM with 10% FBS supplemented with PFD for 24 h. After 24 h of PFD incubation, fresh medium was added, and the cells were stimulated with 12 Gy X‐ray for an additional 24 h. Cell supernatants were collected for ELISA analysis, and the cells were washed twice with PBS and harvested in RIPA buffer for subsequent analysis.

### 2.10 MTT analysis of cell viability

Cells were incubated in the presence of different concentrations of PFD. MTT (3‐[4, 5‐dimethylthiazol‐2‐yl]‐2,5‐diphenyl tetrazolium bromide; 2 mg/ml) was added to the wells and incubated for 3 h at 37°C. The reaction product formazan was extracted with DMSO, and the absorbance was measured at 540 nm as previously described.^21^

### 2.11 Enzyme‐linked immunosorbent assay

The levels of TGF‐β1 (cat. no. DY1679‐05, R&D), CCL2 (cat. no. DY479‐05, R&D) and CXCL1 (cat. no. DY453‐05, R&D) in the cell culture supernatant of the different treatment groups were measured by their respective ELISA kits. The optical density of each sample was measured at 450 nm using a Spectra Max 190 microplate reader.

### 2.12 Flow cytometry

Macrophages in the different treatment groups were digested and washed with PBS three times. Cell suspensions were adjusted to 10^6^/ml. Antibodies against F4/80 (cat. no. 11–480–82, eBioscience) and CD206 (cat. no. 12–2061–82, eBioscience, USA) were added and incubated at room temperature for 30 min, and the cells were analysed by flow cytometry (FACS Verse assay, BD Biosciences).

### 2.13 Immunofluorescence staining of macrophages

Macrophages were cultured in 12‐well plates containing glass slides and were then washed with PBS and fixed with 4% paraformaldehyde for 30 min. After permeabilization with 0.1% Triton X‐100 for 10 min, the specimens were washed with PBS and then blocked with 10% FBS to eliminate nonspecific fluorescence. Immunofluorescence staining was performed using ARG‐1 (1:200, cat. no. ab91279, Abcam), YM‐1 (1:200, cat. no. ab192029, Abcam) and CD163 (1:200, cat. no. 182422, Abcam) primary antibodies, and the cell preparations were incubated with DyLight 488/647‐labelled secondary antibodies (1:200, cat. no. 150077/ab150115, Abcam).

### 2.14 Macrophage migration assay

RAW264.7 cells were seeded into the upper chamber of a Transwell insert (Corning Incorporated) at a density of 6 × 10^4^ cells/well in 200 μl of serum‐free medium and placed on a 24‐well plate containing conditioned medium obtained from nonirradiated or irradiated MLE‐12 cells. After 24 h of incubation, the cell suspension in the upper chamber was aspirated, and the upper surface of the filter was carefully cleaned with cotton buds. Cells that migrated through the polycarbonate membrane were fixed with 70% ethanol for 20 min and stained with 0.5% crystal violet for 15 min. The membrane was cut away from the chamber, and migrated cells on the lower surface of the filter were counted in six representative fields with a microscope at 200× magnification.

### 2.15 Cell co‐culture

MLE‐12 cells were cultured in DMEM supplemented with 10% FBS, cell‐free medium or 50% conditioned medium from RAW264.7 cells treated with PFD (100 µg/ml) and IL‐4 and IL‐13. After 24 h, MLE‐12 cells were washed twice with PBS and harvested in sample buffer as described previously for subsequent Western blot analysis.

### 2.16 Statistical analysis

Unless otherwise indicated, the data are presented as the means ± SD of independent experiments. The statistical significance of the differences between two groups was analysed with Student's *t*‐tests. The calculations were performed using Prism software for Windows (GraphPad Software).

## 3 RESULT

### 3.1 Pirfenidone attenuates pulmonary fibrosis induced by whole‐lung radiation

**FIGURE 1:**
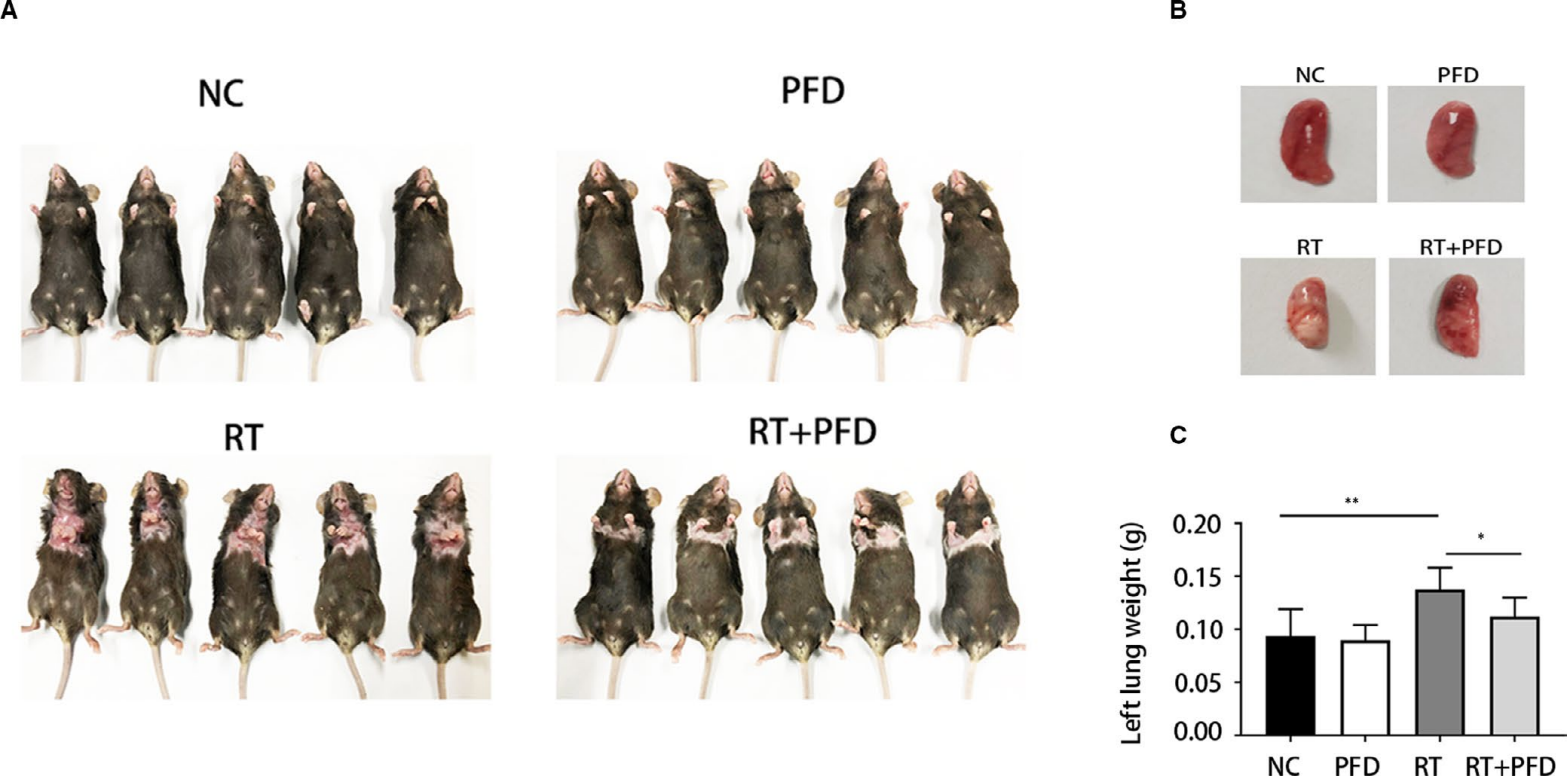
Pirfenidone attenuates pulmonary fibrosis induced by whole‐lung radiation. A, Photographs of mice in the four groups after 84 days of treatment. B, Photographs of the lung tissue in the four groups. C, The mean left lung weights of mice in the four groups (*n* = 5 per group). The values are the means ± SD, *p < 0.05, **p < 0.01

After 50 Gy of whole‐lung irradiation, the skin of mice in the RT and RT + PFD groups showed obvious lesions in the irradiated area, with the colour changing from black to white (Figure 1A). The skin lesions in the RT group were more serious than those in the RT + PFD group. In the RT group, the lung tissues became consolidated and white, with increased weight, and the morphological changes and increased weight were alleviated by PFD treatment (Figure 1B,C).

**FIGURE 2:**
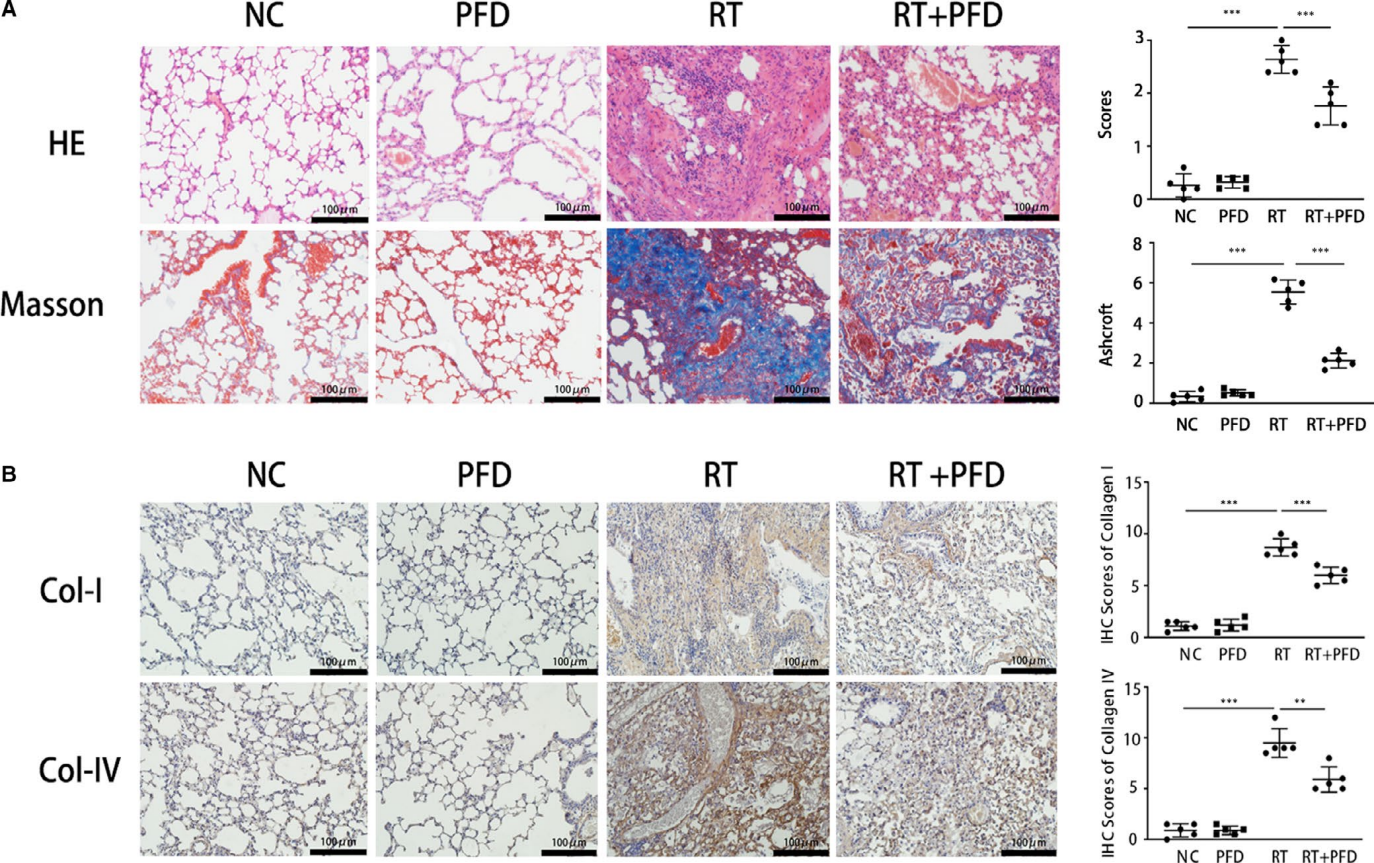
Pirfenidone effectively inhibited alveolar inflammation, pulmonary fibrosis and collagen deposition induced by lung irradiation. A, HE and Masson staining of lung tissues in the four groups and semiquantitative analysis of the degree of pulmonary fibrosis. The values are the means ± SD, **p < 0.01, ***p < 0.001. B, IHC staining of collagen I and collagen IV in lung tissue from the four groups and semiquantitative analysis of collagen I and collagen IV expression. Scale bar = 100 μm. The values are the means ± SD, **p < 0.01, ***p < 0.001

HE staining showed that the alveolar structure of the lung tissue in the NC and PFD groups was clear, with slender alveolar walls and intact capillary walls. In the RT group, the alveolar wall was severely thickened, and the alveolar cavity became obviously small, with many fibroblast aggregates, and patchy fibrosis appeared around blood vessels and the pulmonary interstitial area. After PFD treatment, the histological changes in the lung tissues were significantly alleviated compared with those of the RT group. Masson staining showed that the structure of the lung tissues in the NC and PFD groups was normal. In contrast, the alveolar wall in the RT group showed obvious destruction, with increased collagen deposition (blue area in Figure 2A), while collagen deposition was attenuated in the RT + PFD group (Figure 2A). The expression of collagen I and collagen IV in lung tissue was examined by IHC staining and showed significantly higher expression of collagen I and collagen IV in the RT group than in the NC and PFD groups. The expression of collagen I and collagen IV in the RT + PFD group was obviously decreased (Figure 2B).

### 3.2 Pirfenidone inhibits ionizing radiation‐induced M2 macrophage polarization

**FIGURE 3:**
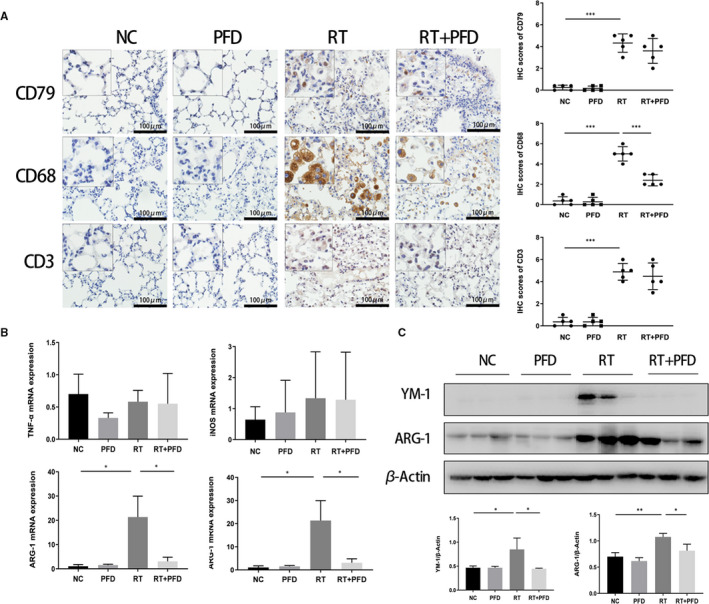
Pirfenidone inhibits ionizing radiation‐induced M2 polarization. A, The expression of CD68, CD79 and CD3 in the lung tissue of mice in the different treatment groups was determined by IHC analysis. Scale bar = 100 μm. B, qRT‐PCR was used to measure the expression of ARG‐1, YM‐1, iNOS and TNF‐α in the lung tissue of mice in the different treatment groups. (*n* = 3 per group). The values are the means ± SD, *p < 0.05, C, Western blotting was used to measure the expression of ARG‐1 and YM‐1 in the lung tissue of mice in the different treatment groups. Data from individual animals (*n* = 3 per group). The values are the means ± SD, *p < 0.05, **p < 0.01

The IHC staining results showed that in the NC and PFD groups, the expression of CD68 (a marker of macrophages), CD79 (a marker of B cells) and CD3 (a marker of T cells) was negative. However, in the RT group, CD68‐positive, CD79‐positive and CD3‐positive immune cells were abundantly distributed in the RILF area. In the RT + PFD group, the proportion of CD68‐positive immune cells decreased significantly in the lung tissue (Figure 3A). The IF staining results showed that the immune cells in the RT group were both CD68‐ and CD163 (a marker of M2 macrophages)‐positive. In the NC and PFD groups, there were no CD68‐ or CD163‐positive cells. In the RT + PFD group, there were fewer CD68‐ and CD163‐positive cells than in the RT group (Figure S1A). Compared with that of the NC and PFD groups, the expression of M2 macrophage‐associated inflammatory factors ARG‐1 and YM‐1 in the RT group was upregulated, as determined by qRT‐PCR. In the RT + PFD group, the expression of ARG‐1 and YM‐1 was downregulated. However, the expression of M1 macrophage‐associated inflammatory factors TNF‐α and iNOS in the four treatment groups was not significantly different (Figure 3B). The expression of these two proteins was the same when analysed by Western blotting (Figure 3C).

### 3.3 Pirfenidone inhibits the polarization of M2 macrophages by downregulating NF‐κB p50

**FIGURE 4:**
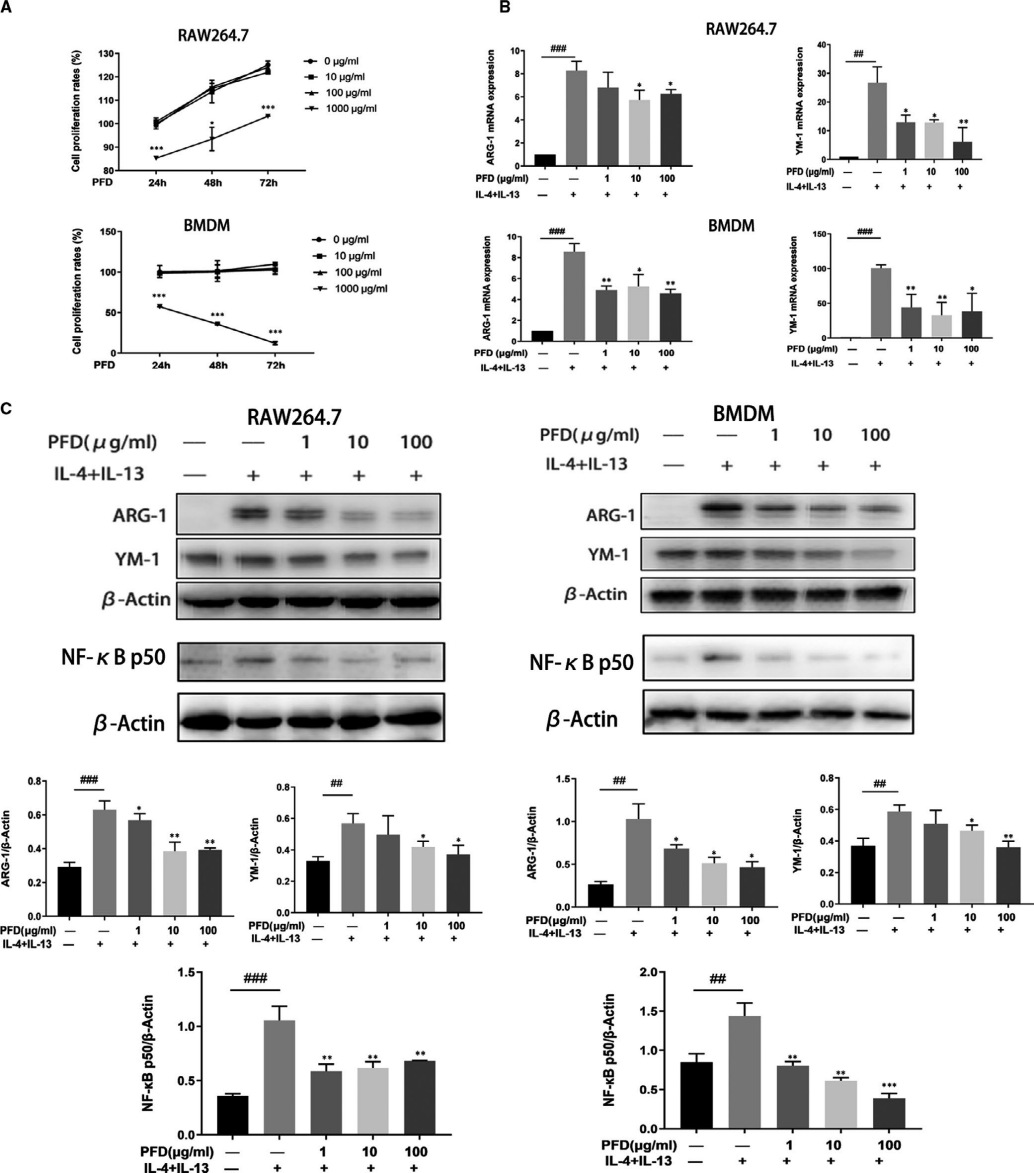
Pirfenidone inhibits the polarization of M2 macrophages by downregulating NF‐κB p50 expression. A, Cytotoxic effects of PFD on RAW264.7 cells and BMDMs. Plots showing the effects of different concentrations of PFD on the growth of RAW264.7 cells and BMDMs during the indicated times. The values are the means ± SD, *p < 0.05, **p < 0.01, when comparing 1000 μg/ml with 0 μg/ml. B, qRT‐PCR was used to analyse the quantitative mRNA expression of the M2 phenotypic markers ARG‐1 and YM‐1 in the 4 different treatment groups of RAW264.7 cells and BMDMs. The values are the means ± SD, ^##^ *p* < 0.01 and ^###^ *p* < 0.001, when compared with the untreated group, *p < 0.05, **p < 0.01, ***p < 0.001, when compared with the IL‐4 + IL‐13–stimulated group. C, Western blots and associated densitometry analysis of the protein expression of the M2 phenotypic markers ARG‐1, YM‐1 and NF‐κB p50 in the 4 different treatment groups of RAW264.7 cells and BMDMs. The values are the means ± SD, ^##^ *p* < 0.01 and ^###^ *p* < 0.001, compared with the untreated group, *p < 0.05, **p < 0.01, ***p < 0.001 compared with the IL‐4 + IL‐13–stimulated group. The tests were repeated in three independent experiments

After RAW264.7 cells were cocultured with BMDMs in the presence of 1000 µg/ml PFD, the proliferation of both cell types was significantly inhibited. PFD at a concentration of 100 µg/ml or less had no effect on the proliferation of either cell type (Figure 4A). PFD at a concentration of 100 µg/ml or less was used to further explore the effect of PFD on the polarity of M2 macrophages. We stimulated macrophages with 10 ng/ml IL‐4 and IL‐13 for 24 h after pretreatment with 100 μg/ml PFD for 24 h. Flow cytometry was used to measure the polarization of F4/80^+^(a marker of macrophages)/CD206^+^(a marker of M2 macrophages) M2 macrophages induced by the different treatments. Flow cytometric analysis showed that the proportion of F4/80^+^/CD206^+^ macrophages was increased in IL‐4– and IL‐13–treated macrophages but decreased after PFD treatment (Figure S2A). qRT‐PCR showed that the mRNA expression of ARG‐1 and YM‐1 in RAW264.7 cells and BMDMs induced by IL‐4 and IL‐13 was inhibited by PFD (Figure 4B). The expression of ARG‐1, YM‐1 and NF‐κB p50 protein in RAW264.7 cells and BMDMs was measured by Western blotting and was also be inhibited by PFD (Figure 4C). In addition, immunocytochemical staining indicated that PFD could significantly inhibit IL‐4– and IL‐13–induced ARG‐1, YM‐1 and CD163 expression (Figure S2B).

### 3.4 Pirfenidone inhibits activation of the TGF‐β1/Smad3 signal pathway in vivo and in vitro

**FIGURE 5:**
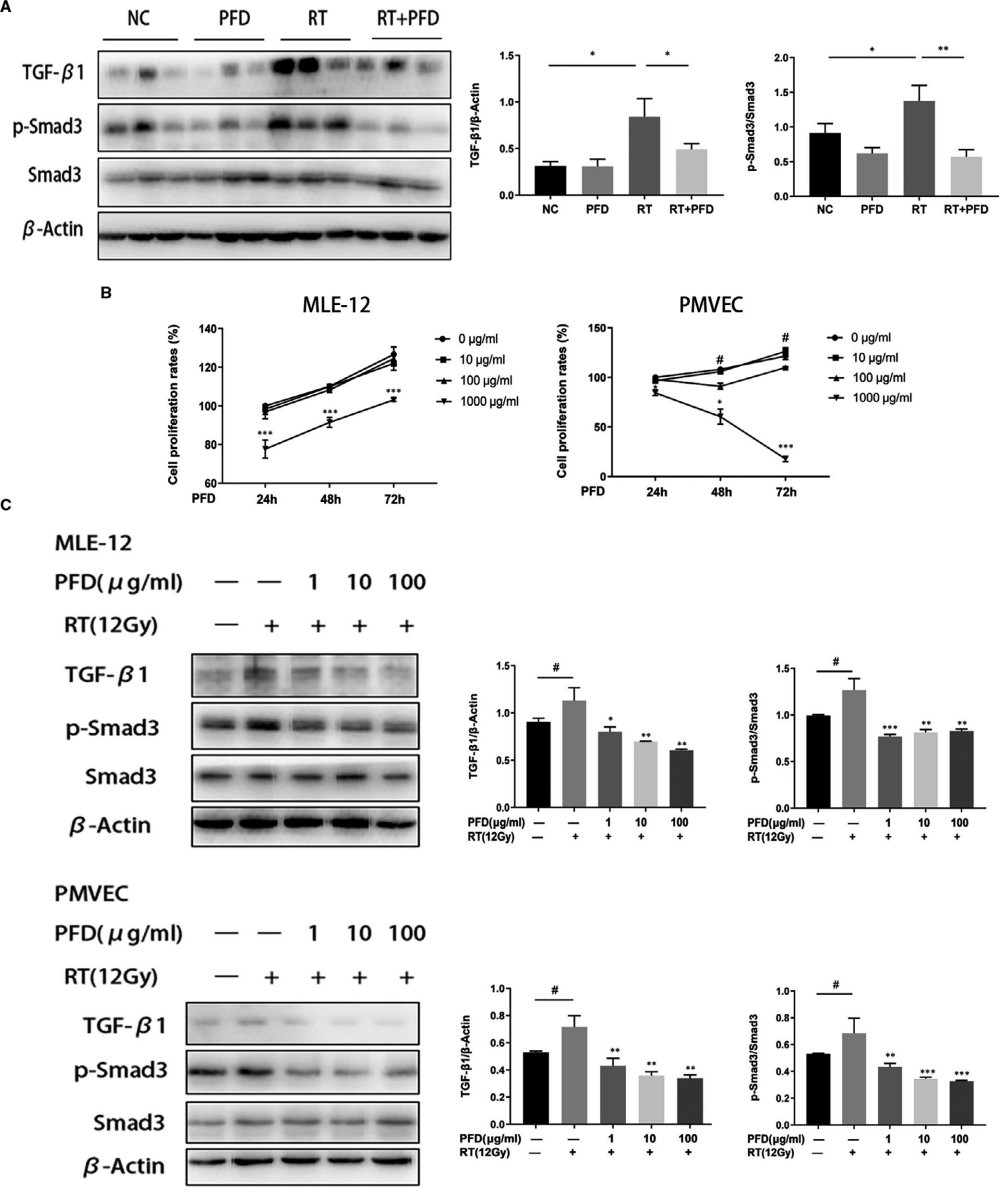
Pirfenidone inhibits irradiation‐induced activation of the TGF‐β1/Smad3 signalling pathway in vivo and in vitro. A, The expression and quantitative analysis of TGF‐β1, p‐Smad3, Smad3 and β‐actin in lung tissue from the four different treatment groups. Data from individual animals (*n* = 3 per group); the values are the means ± SD, *p < 0.05, **p < 0.01. In Figures 3C and 5A, we run the same four subgroups, NC, PFD, RT, RT + PFD and stain YM‐1, ARG‐1 (in Figure 3C) and TGF‐β1, p‐Smad3, Smad3 (in Figure 5A). Therefore, the β‐actin in Figure 3C and Figure 5A is the same band. B, Cytotoxic effects of PFD on the proliferation of MLE‐12 cells and PMVECs. Plots showing the effects of different concentrations of PFD on the growth of MLE‐12 cells and PMVECs over the indicated times. The values are the means ± SD. MLE‐12 cells: ***p < 0.001, 1000 μg/ml compared with 0 μg/ml PMVECs: **p < 0.05, ***p < 0.001, when comparing 100 μg/ml with 0 μg/ml; ^###^ *p* < 0.001, when comparing 1000 μg/ml with 0 μg/ml. C, The expression and quantitative analysis of TGF‐β1, p‐Smad3, Smad3 and β‐actin in MLE‐12 cells and PMVECs after RT and PFD treatment. The values are the means ± SD, ^#^ *p* < 0.05, compared with the untreated group, *p < 0.05, **p < 0.01, ***p < 0.001, compared with the IL‐4 + IL‐13–stimulated group. The tests were repeated in three independent experiments

The protein expression of TGF‐β1 and p‐Smad3 in lung tissues was measured by Western blotting and showed that in the RT + PFD group (Figure 5A), the RT‐induced expression levels of TGF‐β1 and p‐Smad3 were downregulated by PFD (the β‐actin in Figure 3C and Figure 5A is the same band, because we run the same four subgroups). The cytotoxicity analysis showed that 1000 µg/ml PFD for more than 48 h significantly inhibited the proliferation of MLE‐12 cells and that 100 μg/ml PFD for more than 48 h significantly inhibited the proliferation of PMVECs. Therefore, MLE‐12 cells and PMVECs were stimulated with 100 μg/ml PFD for no more than 24 h for the following experiments (Figure 5B). After MLE‐12 cells and PMVECs were subjected to 12 Gy irradiation, the expression of TGF‐β1 and p‐Smad3 was upregulated. TGF‐β1 and p‐Smad3 expression in MLE‐12 cells and PMVECs was downregulated after pretreatment with PFD for 24 h in vitro (Figure 5C).

### 3.5 Pirfenidone is involved in the crosstalk between alveolar epithelial cells and macrophages

**FIGURE 6:**
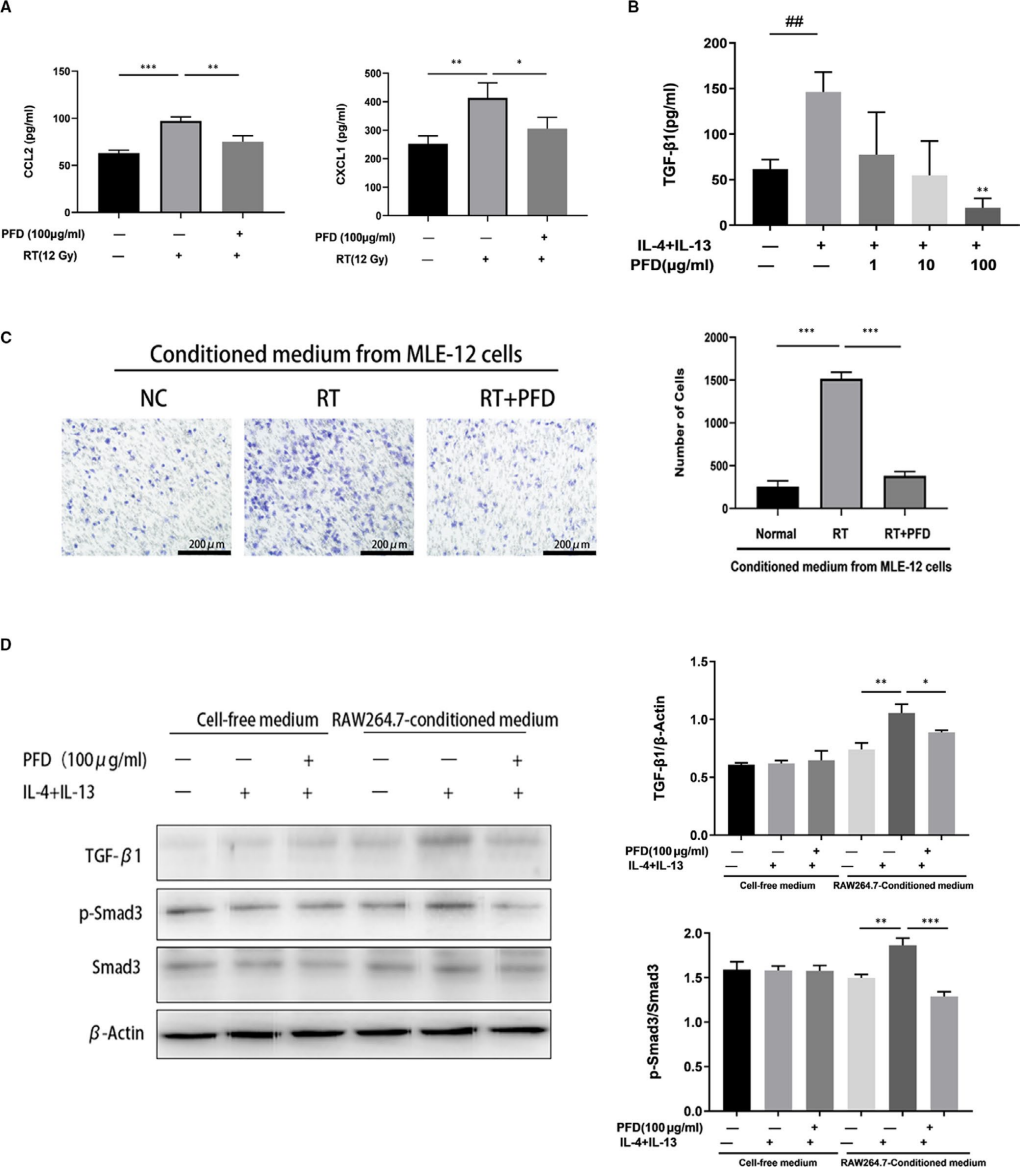
Pirfenidone is involved in the crosstalk between alveolar epithelial cells and macrophages. A, The protein levels of CCL2 and CXCL1 in MLE‐12 cell culture supernatants were detected by ELISA after irradiation with 4 different doses. The values are the means ± SD, *p < 0.05, **p < 0.01, compared with 0 Gy. B, The protein expression of TGF‐β1 in culture supernatants of RAW264.7 cells in the groups treated with IL‐4 + IL‐13 and PFD was measured by ELISA. The values are the means ± SD, **p < 0.01, compared with 0 Gy. C, The migration of RAW264.7 cells was assessed in a Transwell assay. Representative images (left) and statistical analysis (right) show the migration of RAW264.7 cells induced by different conditioned media. Scale bar = 200 μm. The values are the means ± SD, ***p < 0.001. D, The expression levels of p‐Smad3, Smad3 and β‐actin in MLE‐12 cells cultured in conditioned media collected from RAW264.7 cells in the different treatment groups were analysed by Western blotting. The values are the means ± SD, *p < 0.05, **p < 0.01, ***p < 0.001. The tests were repeated in three independent experiments

The expression of granulocyte‐macrophage colony‐stimulating factor (GM‐CSF), chemokine (C‐C motif) ligand 2 (CCL2), chemokine (C‐X‐C motif) ligand 1 protein (CXCL1), CXCL5, CXCL10 and CXCL16 in MLE‐12 cells was evaluated at 24 h after irradiation with increasing doses of X‐ray (Figure S3A). The secretion of CCL2 and CXCL1 in the culture supernatant of MLE‐12 cells was also measured at 24 h after irradiation (Figure S3B), and the secretion of CCL2 and CXCL1 was inhibited by PFD treatment (Figure 6A). In addition, the Transwell assay showed that the migration of RAW264.7 cells induced by the conditioned medium of irradiated MLE‐12 cells was also inhibited by PFD treatment in vitro (Figure 6C).

Transforming growth factor‐β1 expression in the culture supernatants of RAW264.7 cells was significantly elevated after induction with IL‐4 and IL‐13. The expression of TGF‐β1 was also inhibited by PFD, and the expression was lower with increasing concentrations of PFD (Figure 6B). The protein expression of TGF‐β1 and p‐Smad3 in MLE‐12 cells was significantly elevated after culture with the supernatant of RAW264.7 cells stimulated with IL‐4 and IL‐13 for 24 h, and these effects were inhibited by PFD treatment. The expression of TGF‐β1 and p‐Smad3 in MLE‐12 cells did not change in cell‐free medium with or without IL‐4 and IL‐13 and PFD (Figure 6D). Taken together, these results demonstrated that PFD inhibited not only macrophage infiltration induced by chemokines secreted by irradiated MLE‐12 cells but also macrophage production of TGF‐β1, which could affect the activation of TGF‐β1/Smad3 in MLE‐12 cells.

## 4 DISCUSSION

Radiotherapy plays a very important role in the treatment of thoracic tumours. It is critical to find effective drugs to treat RP and reduce the risk of RILF. Clinically, corticosteroids are mainly used to treat RP, and early intervention is expected to lead to recovery. Although the risk of RILF can be predicted by measuring many blood biochemical indexes, such as TGF‐β, IL‐6, Krebs von den Lungen‐6 (KL‐6), surfactant proteins and interleukin‐1 receptor antagonist (IL‐1ra),^22^ most drugs for treating RILF are still in preclinical research. There are essentially no effective treatments for RILF. In the present study, we used a mouse model of RILF to investigate the antifibrotic effect of PFD. RILF is a slow progressive process that generally occurs 6–18 months after radiation in both animals and humans,^23^, ^24^ and 15–20 Gy whole‐lung X‐ray irradiation of mice is a common method to establish an RILF mouse model.^25^, ^26^, ^27^ However, in our study, the mice were irradiated with 50 Gy by a small animal radiation research platform (SARRP), which is different from other small animal irradiation systems in that it satisfies all of the following requirements: high dose rate, small beam diameter and accurate dose location based on image guidance.^28^ It has been reported that SARRP can improve the overall survival rate of mice by reducing lung side effects after high‐precision heart irradiation.^29^ Fibrosis could be seen in the lungs of mice irradiated with 30 Gy, while overt and intense fibrosis could be seen after irradiation with 60 Gy and 90 Gy.^30^ Similarly, our results showed that pulmonary fibrosis in mice was severe at 150 days after 50 Gy irradiation, while orally administered 300 mg/kg PFD significantly attenuated pulmonary inflammatory infiltration and collagen accumulation. These antifibrotic effects are consistent with the results of different animal fibrosis studies.^18^, ^31^

Using a prototypical model of RILF, we confirmed that the administration of PFD could significantly inhibit ionizing radiation‐induced activation of the TGF‐β1/Smad3 signalling pathway. In vitro, irradiation‐induced activation of the TGF‐β1/Smad3 signalling pathway was significantly inhibited by PFD at concentrations less than 100 μg/ml without cytotoxicity. This finding is consistent with a recent report that PFD has a wide range of antifibrotic effects, including the inhibition of TGF‐β1/Smad3 signalling.^32^, ^33^

A large number of animal experiments have shown that the occurrence of RILI is mainly caused by a series of pathophysiological reactions that interact with a variety of damaged cells and are regulated by a variety of cytokines.^34^ Macrophages play a key role in the development of RILI because they are the first line of defence against external invasion.^35^ The release of M2 cytokines has been reported to contribute to the excessive repair of damaged tissue and accelerate fibrosis, such as that induced by parasitic infections,^36^ fungal infections^37^ and bleomycin.^38^ M2 cytokines play the same role in radiation‐induced fibrosis in tissues or organs, such as the skin,^39^, ^40^ intestine^41^and lung.^42^ In our study, the recruitment of macrophages to the lungs of thoracically irradiated mice increased significantly, and most of them were M2 macrophages, which highly expressed ARG‐1 and YM‐1. PFD inhibited the polarization of M2 macrophages, which was characterized by a decrease in the M2 ratio and ARG‐1 and YM‐1 levels in vivo and in vitro. A previous study identified the p50 subunit of NF‐κB as a key regulator of M2 macrophage polarization,^43^, ^44^ and the downregulation of NF‐κB p50 expression may be one of the mechanisms by which PFD inhibits the polarization of M2 macrophages.

We confirmed that macrophages cultured with IL‐4 and IL‐13 secreted high levels of TGF‐β1, while PFD could significantly inhibit the expression of TGF‐β1 by M2 macrophages, which was consistent with the findings of Toda M et al.^45^ AECs can activate fibroblasts through epithelial‐mesenchymal transition (EMT) and then differentiate into myofibroblasts, which form characteristic fibroblast foci and secrete extracellular matrix (ECM), leading to fibrosis. It has been reported that M2 macrophages but not M1 macrophages can induce EMT in AECs, and this process is mainly regulated by the TGF‐β1/Smad signalling pathway.^46^, ^47^ Our current study showed that only conditioned medium from RAW264.7 cells treated with IL‐4 and IL‐13 increased the expression of TGF‐β1 and p‐Smad3, and the same conditioned medium from RAW264.7 cells that had been treated with PFD (100 µg/ml) significantly suppressed TGF‐β1 and p‐Smad3. These findings suggested that PFD‐induced inhibition of TGF‐β1 secretion by M2 macrophages prevents pulmonary fibrosis by inhibiting the TGF‐β1/Smad3 pathway in AECs.

Irradiated AECs can secrete a large amount of chemokines, such as CCL5, CCL2 and GM‐CSF,^47^ which recruit inflammatory monocytes and neutrophils to the injured site. Our results showed that PFD could significantly inhibit the expression of multiple chemokines induced by ionizing radiation in MLE‐12 cells. It has been reported that PFD reduces macrophage infiltration in nephrectomized rats^48^ and inhibits both IL‐17A–induced macrophage migration and MCP‐1–induced macrophage migration in vitro.^49^ We hypothesize that PFD inhibits macrophage infiltration by reducing the secretion of chemokines from irradiated MLE‐12 cells. Consistent with this hypothesis, we cultured macrophages with the supernatant from irradiated MLE‐12 cells in Transwells. We found that the migration of macrophages was significantly enhanced in the presence of conditioned medium from irradiated MLE‐12 cells, and PFD could impair macrophage migration to irradiated MLE‐12 cell.

We have to admit that there is a deficiency in our study: although a number of animal studies have pointed out that CMC has no effect on histopathological and biochemical changes,^50^, ^51^, ^52^ compared with normal saline, the mice in NC group using 0.5% CMC as control would be a better choice.

**FIGURE 7:**
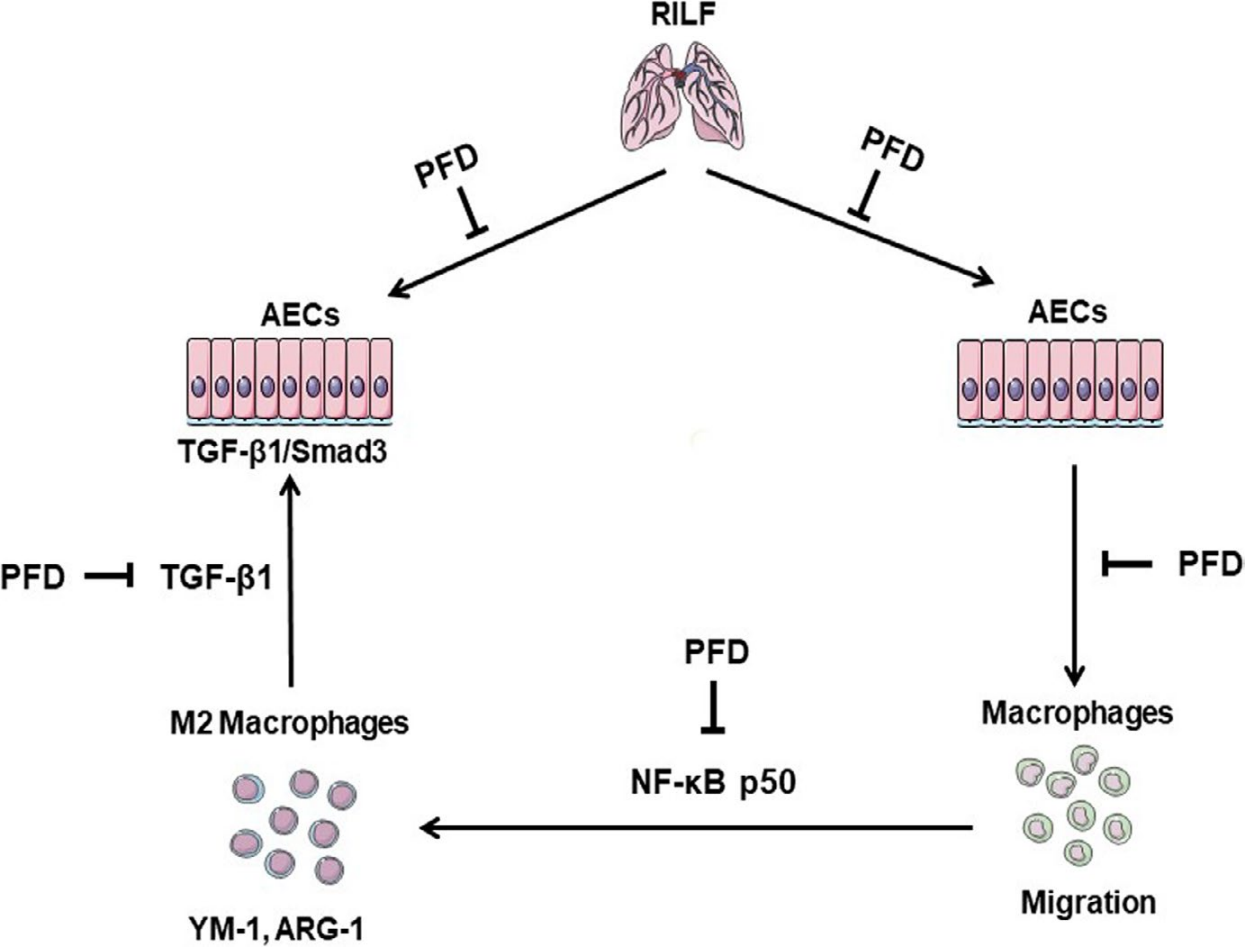
The working model of the preventive and therapeutic effects of PFD on RILF. PFD has a significant inhibitory effect on pulmonary fibrosis and fibrosis in other organs and is a broad‐spectrum antifibrosis drug. In our study, we show that PFD protects against RILF by reducing the recruitment of M2 macrophages and inhibiting activation of the TGF‐β1/Smad3 signalling pathway. PFD is also involved in the crosstalk between macrophages and AECs: 1. PFD reduces the migration of macrophages by inhibiting the secretion of chemokines by MLE‐12 cells induced by ionizing radiation. 2. PFD inhibits activation of the TGF‐β1/Smad3 signalling pathway in MLE‐12 cells by inhibiting the secretion of TGF‐β1 by M2 macrophages. Here, we provide a new theoretical basis for the use of PFD in the treatment of RILF. Parts of the figure were drawn using images from Servier Medical Art (https://smart.servier.com/).

In conclusion, our findings indicated that PFD is a potential therapeutic agent to ameliorate fibrosis in RILF by reducing M2 macrophage infiltration and inhibiting the activation of TGF‐β1/Smad3 signalling (Figure 7). A pilot study confirmed that PFD is effective in ameliorating the disability associated with RILF.^53^ Our cancer centre is also conducting a multicentre phase II clinical trial (NCT03902509) on the treatment of RILI with PFD. We believe that PFD will be beneficial RILF patients in the near future.

## CONFLICT OF INTEREST

None.

## AUTHOR CONTRIBUTIONS

**Hangjie Ying:** Data curation (lead); Funding acquisition (equal); Writing‐original draft (lead). **Min Fang:** Formal analysis (equal); Funding acquisition (equal); Methodology (equal). **Qing Qing Hang:** Data curation (equal); Methodology (equal). **Yamei Chen:** Data curation (equal); Methodology (equal). **Xu Qian:** Investigation (supporting); Project administration (supporting). **Ming Chen:** Project administration (lead).

## Supporting information

Fig S1

Fig S2

Fig S3

Supplementary Material
