# Supplementary figures and images for: Pirfenidone modulates macrophage polarization and ameliorates radiation‐induced lung fibrosis by inhibiting the TGF‐β1/Smad3 pathway
Source: J Cell Mol Med. 2021 Jul 29;25(18):8662–75. doi: 10.1111/jcmm.16821 (PMC8435416; doi:10.1111/jcmm.16821)

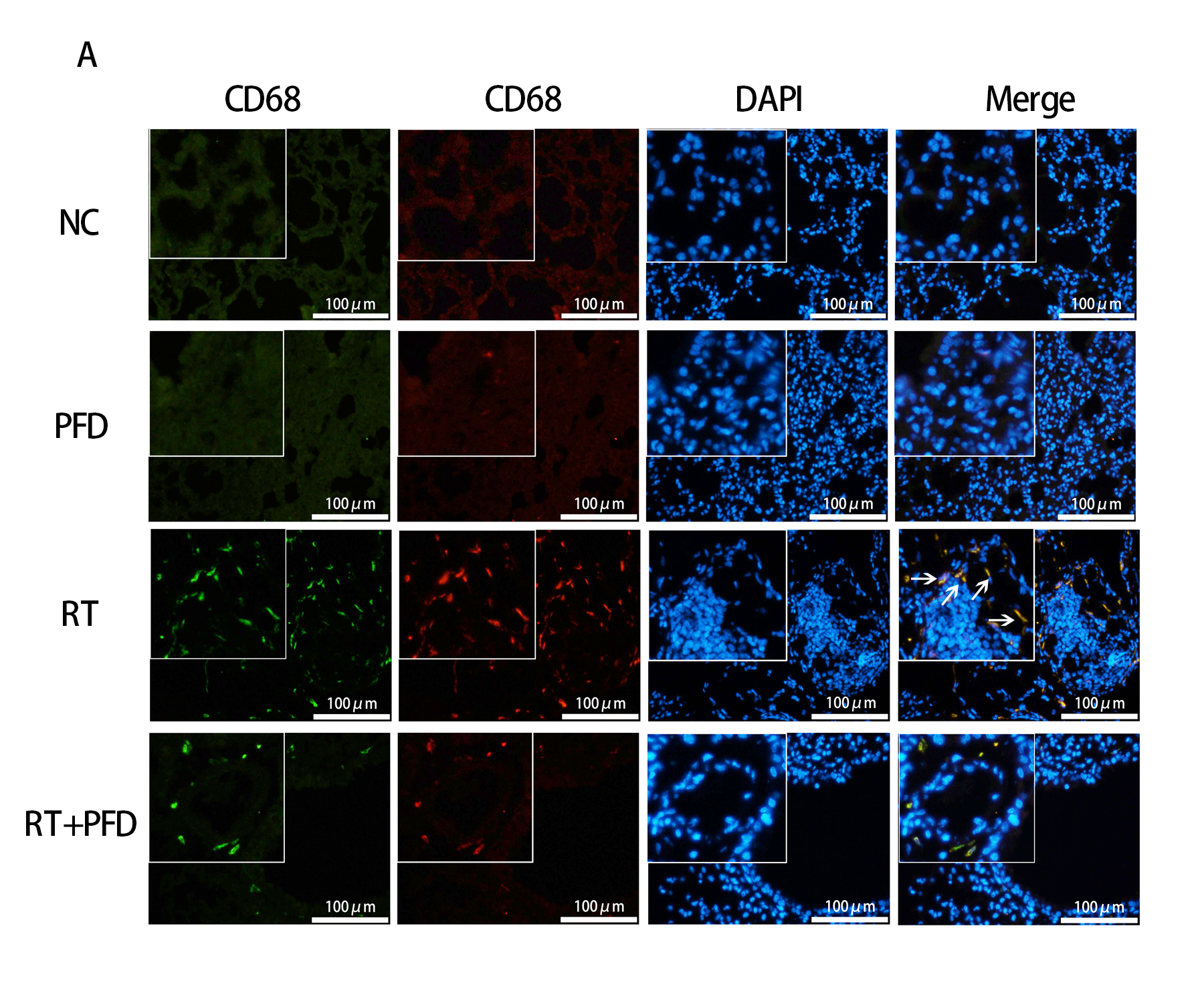

Supplement: Supplementary file 1 — Fig S1 [file JCMM-25-8662-s003.tif]

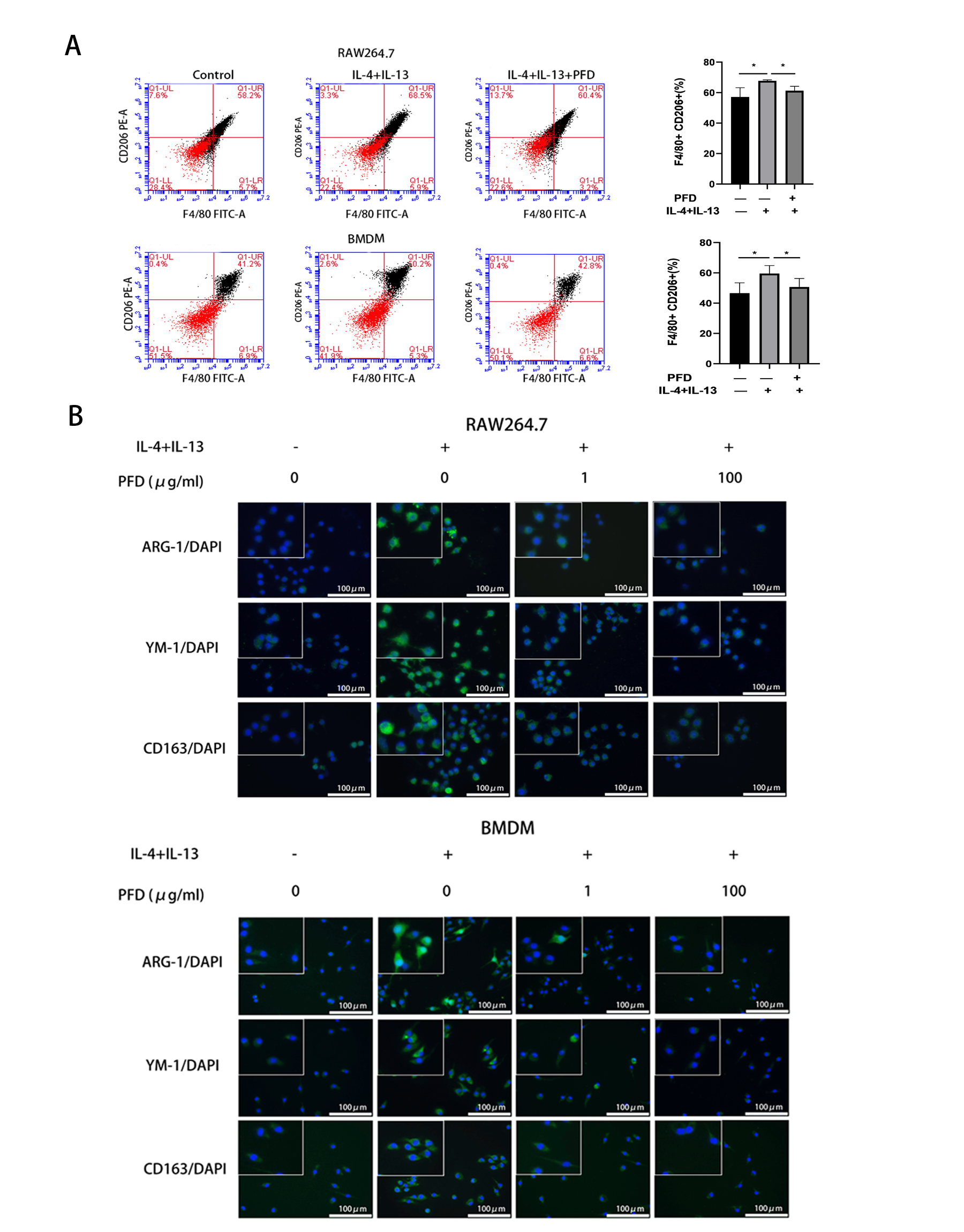

Supplement: Supplementary file 2 — Fig S2 [file JCMM-25-8662-s002.tif]

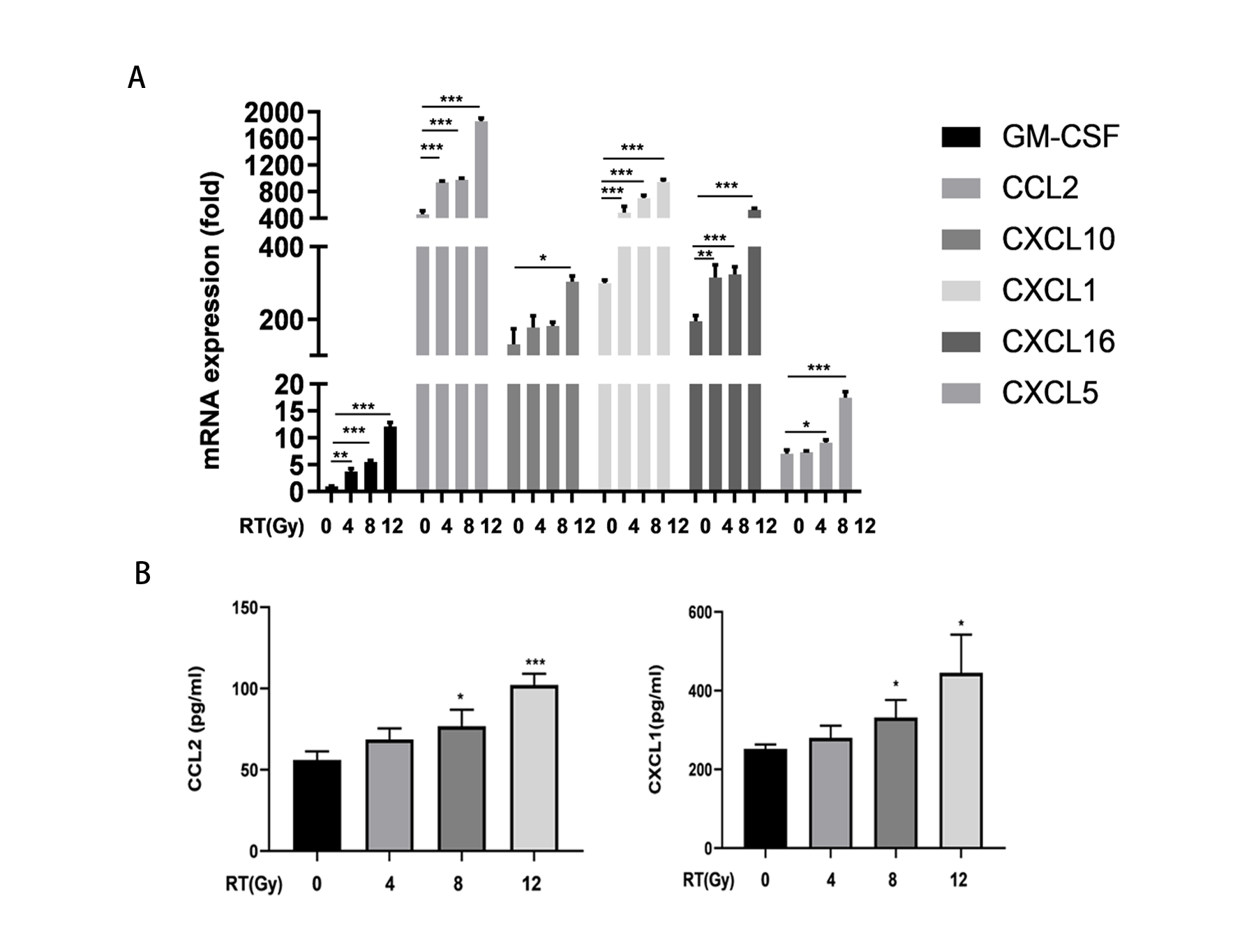

Supplement: Supplementary file 3 — Fig S3 [file JCMM-25-8662-s004.tif]
