# Supplementary material for: Pirfenidone modulates macrophage polarization and ameliorates radiation‐induced lung fibrosis by inhibiting the TGF‐β1/Smad3 pathway
Source: J Cell Mol Med. 2021 Jul 29;25(18):8662–75. doi: 10.1111/jcmm.16821 (PMC8435416; doi:10.1111/jcmm.16821)
Supplement: Supplementary file 4 — Supplementary Material [file JCMM-25-8662-s001.docx]

Supplementary Figures

Supplementary Figure 1.PFD inhibits ionizing radiation-induced M2 polarization.

1. Lung sections were fixed and stained with anti-CD68 (green) and anti-CD163 (red). Arrows point to cells that are CD68+CD163+. Scale bar = 100 μm.

Supplementary Figure 2.PFD inhibits the polarization of M2 macrophages in vitro

A. Flow cytometry and quantitative analysis was used to analyze the proportion of F4/80+/CD206+ macrophages in the 4 different treatment groups of RAW264.7 cells and BMDMs. The values are the means ±SD, *p<0.05.

B. The expression of ARG-1, YM-1 and CD163 was determined by immunofluorescence staining in 4 different treatment groups of RAW264.7 cells and BMDMs. Scale bar = 100 μm.

Supplementary Figure 3. Ionizing radiation increases the expression of chemokines in alveolar epithelial cells.

1. The mRNA expression of GM-CSF, CCL2, CXCL1, CXCL5, CXCL10 and CXCL16 was measured at 24 h after irradiation with 0 to 12 Gy. The values are the means±SD, *p<0.05, **<0.01, ***p<0.001.
2. The secretion of CCL2 and CXCL1 by MLE-12 cells was measured by ELISA after different treatments with PFD and radiation. The values are the means±SD, *p<0.05, **<0.01.The tests were repeated in three independent experiments
